# Role of Inflammation in Diabetic Retinopathy

**DOI:** 10.3390/ijms19040942

**Published:** 2018-03-22

**Authors:** Anne Rübsam, Sonia Parikh, Patrice E. Fort

**Affiliations:** 1 Department of Ophthalmology and Visual Sciences, University of Michigan, Ann Arbor, MI 48105, USA; aruebsam@med.umich.edu (A.R.); soniapar@umich.edu (S.P.); 2 Department of Molecular and Integrative Physiology, University of Michigan, Ann Arbor, MI 48105, USA

**Keywords:** diabetic retinopathy, neuroprotection, inflammation, neurodegeneration, pathophysiology, microglia, astrocytes, Müller glial cells, crystallins

## Abstract

Diabetic retinopathy is a common complication of diabetes and remains the leading cause of blindness among the working-age population. For decades, diabetic retinopathy was considered only a microvascular complication, but the retinal microvasculature is intimately associated with and governed by neurons and glia, which are affected even prior to clinically detectable vascular lesions. While progress has been made to improve the vascular alterations, there is still no treatment to counteract the early neuro-glial perturbations in diabetic retinopathy. Diabetes is a complex metabolic disorder, characterized by chronic hyperglycemia along with dyslipidemia, hypoinsulinemia and hypertension. Increasing evidence points to inflammation as one key player in diabetes-associated retinal perturbations, however, the exact underlying molecular mechanisms are not yet fully understood. Interlinked molecular pathways, such as oxidative stress, formation of advanced glycation end-products and increased expression of vascular endothelial growth factor have received a lot of attention as they all contribute to the inflammatory response. In the current review, we focus on the involvement of inflammation in the pathophysiology of diabetic retinopathy with special emphasis on the functional relationships between glial cells and neurons. Finally, we summarize recent advances using novel targets to inhibit inflammation in diabetic retinopathy.

## 1. Introduction

Diabetic retinopathy (DR) is the primary cause of visual impairment in the working-age population of the Western world [1]. Among microvascular complications related to diabetes mellitus such as nephropathy and neuropathy, DR is the most common. The prevalence rate for DR for all adults with diabetes aged 40 and older is 28.5% in the United States (4.2 million people) while estimated at 34.6% worldwide (93 million people) [2,3]. With the prevalence of diabetes expected to continue to rise, the prevalence of DR in the United States by year 2020 is expected to be 6 million persons with 1.34 million persons having vision-threatening disease [4]. The substantial worldwide public health burden of DR highlights the importance of continuously searching for new approaches beyond current standards of diabetes care.

**Figure 1:**
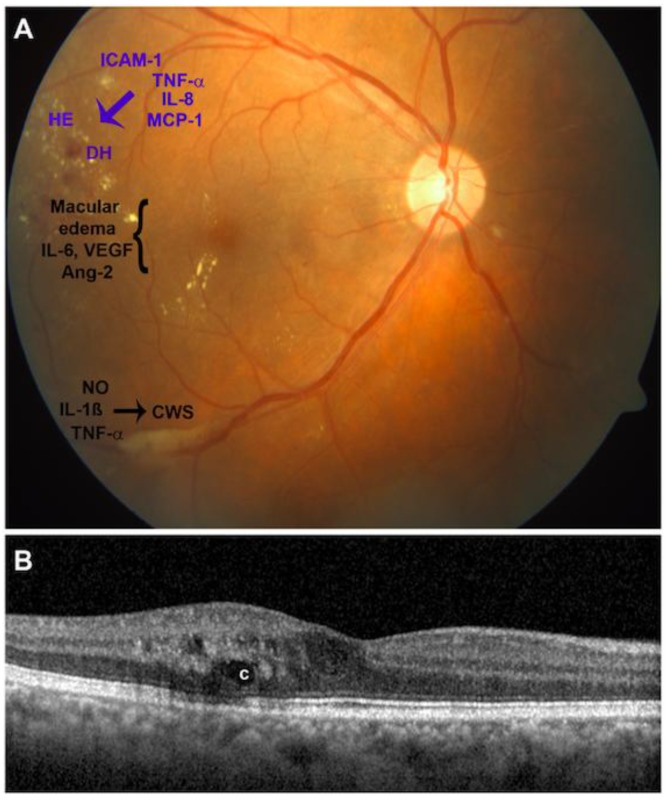
Clinical features of diabetic retinopathy and causative pro-inflammatory chemokines. (**A**) A fundus photograph shows the right eye of a 57-year-old man with 20/80 visual acuity and signs of severe non-proliferative diabetic retinopathy with non-significant macular edema (the region of macular edema is indicated by the bracket). Vascular pathologies are depicted in blue, whereas neurodegenerative features are black. Notable features include dot blot hemorrhages, DH; hard exudates, HE; cotton-wool spots, CWS. Each are associated with the upregulation of certain chemokines; (**B**) Optical coherence tomography with a horizontal scan through the central fovea reveals moderate thickening and edema of the macula with cysts; (**C**) Ang: Angiopoietin; IL: interleukin; TNF: Tumor Necrosis Factor; NO: nitric oxide; MCP: Monocyte Chemoattractant Protein.

Diabetic retinopathy is categorized based on the presence of ophthalmoscopically visible vascular (and closely associated) lesions. It is staged into a non-proliferative stage, characterized by vascular tortuosity, retinal hemorrhages, microaneurysms and lipid exudates; and a proliferative stage, where fragile new aberrant vessels develop (Figure 1). An important additional categorization in DR is diabetic macular edema (DME), a fluid accumulation into the neural retina, which leads to abnormal retinal thickening and often cystoid edema of the macula. DME could occur across all DR severity levels, of both NPDR and PDR and represents the most common cause of vision loss in patients with DR. While for diabetes the breakdown of the inner blood retinal barrier (BRB) is believed to play a dominant role in vascular leakage [5,6], an important role of the outer BRB has recently gained momentum (for review [7]).

Clinical evidence indicates that there is a combination of capillary occlusion and an increased capillary permeability. The ability of vascular endothelial growth factor (VEGF) to promote both vascular permeability and angiogenesis made it a likely contributor to the vascular dysfunctions observed in severe DR [8,9]. Although significant success could be demonstrated with anti-VEGF therapy, a number of limitations exist ranging from the need for repeated intraocular injections, its efficacy only for late stages of the diseases, and most importantly the fact that only approximately half of the patients respond to the treatment [10].

With advancement in diagnostic tools, it has been demonstrated that patients with diabetes exhibit early signs of altered neuroretinal function even before the appearance of microvascular lesions [11]. Currently, there are still no therapies targeting these early stages of the disease to prevent alterations of the neuroretina and thereby preserve visual function. In the past decade, a variety of physiologic and molecular changes consistent with a role of inflammation have been found in the retinas or vitreous humor of diabetic animals and patients [12,13,14,15,16,17,18]. While the detailed mechanisms remain to be specified, these inflammatory changes seems to play a key role important in the development of DR as their inhibition has been shown to impact the development of retinal alterations in animal models of diabetes [19]. Studies using anti-inflammatory agents such as salicylates or minocycline in patients with DR gave further evidence that regulating the inflammatory response can be beneficial to prevent irreversible vascular and neuronal perturbations over time [20,21].

In this review, we analyze evidence that supports the hypothesis, that inflammatory-like processes play a critical role in the development of the early and late stages of the retinopathy with special emphasis on the role of inflammation in the impaired neuro-glial crosstalk in DR.

## 2. Emergence of the Neurovascular Unit and Implications for Early DR

Many DR studies, both clinical and using animal models focused on vascular dysfunction, such as impaired endothelial cells, death of pericytes, thickening of retina capillary basement membrane and altered tight junctions. However, diabetic microvasculopathy does not explain the susceptibility of peripheral nerves, cerebral complications or the early loss of retinal function. Beginning in the late 1990s, the concept of the neurodegenerative aspect of DR started to emerge. Several excellent reviews published in the last decade have summarized the evidence that neurons are vulnerable and die early in diabetic retinopathy, however, the pathophysiological mechanisms underlying this neurodegenerative process remain to be clearly elucidated [22,23]. While retinal ganglion cells (RGCs) are the earliest cells affected and have the highest rate of apoptosis [24,25]. An elevated rate of apoptosis has been also observed in the outer nuclear layer, with a reduction in photoreceptors seen between 4 and 24 weeks after diabetes onset [26,27]. Consistent with this, molecular analyses revealed that proteins important for photoreceptor function, such as rhodopsin, were affected before the appearance of microangiopathy in diabetes [28].

In addition to neuronal cell death, another early feature of diabetic-induced retinal neurodegeneration that has recently gained traction is glial cell activation and dysfunction. Müller glial cells (MGCs), the main glial cells of the retina, play a central role in retinal metabolism, making them highly sensitive to metabolic alterations such as those associated with diabetes. One of the early signs of retinal metabolic stress is the upregulation of glial fibrillary acidic protein (GFAP) by MGCs, an observation classically reported in animal models as well as in tissues from diabetic patients with no to mild NPDR [29,30]. Consequently, the resident immune cells in the retina, called microglia, also become activated and start to produce pro-inflammatory mediators, exacerbating neuro-glial and vascular dysfunction [31].

**Figure 2:**
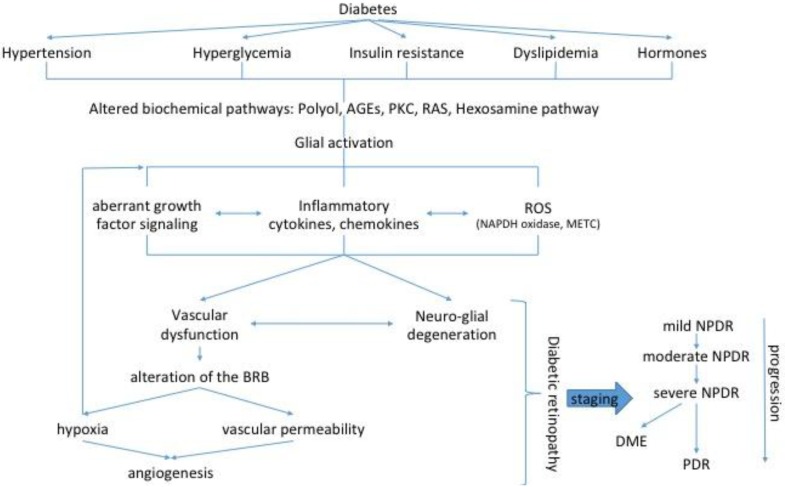
Schematic representation of pathogenic mechanisms leading to sight-threatening endpoints of diabetic retinopathy (DR): proliferative DR (PDR) and diabetic macular edema (DME). Metabolic alterations are first sensed by glial cells resulting in glial dysfunction, which induces inflammation, aberrant signaling of trophic factors and metabolic dysregulation all leading to neuronal apoptosis. Neurodegeneration also participate in blood–retinal barrier (BRB) breakdown, vasoregression and consecutive hypoxia, the main features of early microvascular abnormalities and the end stage neovascularization. According to the vascular lesions DR is staged by ophthalmoscopy in several stages of severity. AGE, advanced glycation end-products; PKC, protein kinase C; RAS, renin–angiotensin system, ROS, reactive oxygen species; NADPH, Nicotinamide adenine dinucleotide phosphate; mETC, mitochondrial electron transport chain; NPDR, non-proliferative DR.

Those retinal abnormalities in neurons and glial cells are believed to drive a variety of functional changes that often precede clinically visible vascular lesions in DR. Among those functional changes are deficits pointing to ganglion cells such as the pattern electroretinogram (ERG), altered microperimetric [32] and perimetric testing [11]; or more generally the inner retina such as increased implicit times and reduction in oscillatory potentials in the multifocal ERG (mfERG) [33]. Other less cellular specific alterations have also been reported including abnormal dark adaptation, contrast sensitivity [34] and color vision [35]. Optical coherence tomography (OCT) is a widely utilized method for imaging the individual neural layers of the retina in many ophthalmic conditions. Several measures associated with OCT imaging are being explored as potential neuroretinal biomarkers of visual defects in eyes with DME and NPDR. Among those that are currently being assessed are ganglion cell/nerve fiber layer thinning, disorganization of the retinal inner layers, and photoreceptor disruption [36,37,38]. The latest progress in this domain is the technique of OCT angiography, which permits to combine with the anatomical observation the non-invasive study of the different layers of retinal microvasculature and choroid circulation via motion contrast imaging [39]. All these observations strongly suggest that diabetes directly affects the neuroretina rather than being solely secondary to the breakdown of the blood-retinal barrier (BRB). Several interconnected biochemical pathways, activated during diabetes, increase the expression of angiogenic and inflammatory mediators and induce aberrant growth factor signaling, all of which have been directly linked to neurodegeneration and vascular dysfunction (Figure 2).

Altogether these new insights into retinal physiology have led to the emergence of the concept that the retinal dysfunction associated with diabetes may be viewed as a change in the retinal neurovascular unit [40]. This concept of the neurovascular unit really emphasizes the intimate relationship between retinal neurons, (photoreceptors, horizontal and bipolar cells, amacrine and ganglion cells), their supporting cells (astrocytes and Müller glial cells), and the vascular beds (endothelial cells and pericytes). Proper function of all the elements of this unit is essential for normal retinal function as it allows the neural retina to adapt to varying physiological conditions, therefore, new therapeutic approaches promoting a “healthy” unit should be targeted.

## 3. Role of Inflammation in Diabetic Retinopathy

### 3.1. Inflammation

Inflammation is a non-specific response to injury or stress that includes a variety of functional and molecular mediators. When invading pathogens are involved, they are recognized by pattern recognition receptors, such as Toll-like receptors (TLRs) and the receptor of advanced glycation end products [36]. The molecules of the pathogens that bind to these receptors are called pathogen-associated molecular patterns (PAMPs) [41]. TLR activation or, in absence of pathogens, tissue stress alone, can lead to de-inhibition of the transcription factor Nuclear factor kappa B (NF-κB), which translocates into the nucleus to stimulate transcription of pro-inflammatory cytokines, acute phase proteins and chemokines, such as IL-6, TNF-α, IL-1β, and monocyte chemoattractant protein-1 (MCP-1). All these pro-inflammatory chemokines play a major role in the recruitment and activation of monocytes and leukocytes and the subsequent inflammatory responses. Inflammation normally resolves promptly through a coordinated program that includes resolvins, lipoxins and protectins [42]. When not resolved in a timely fashion the typically beneficial effect of inflammation is lost and dramatic consequences ensues [43].

### 3.2. Evidences of Inflammation in Diabetic Retinopathy Pathogenesis

Increasing evidence points to inflammation as a critical contributor to the development of DR [44,45,46]. Many inflammatory cytokines and chemokines are increased in serum and ocular samples (vitreous and aqueous humor) from diabetic patients with DR (Table 1).

Glial activation, or gliosis has been suggested to happen in patients with no clinical signs of DR. Increased levels of GFAP has indeed been reported in the aqueous humor of such patients [47]. Since Müller glial cells are a significant source of numerous factors including inflammatory modulators, this suggests that retinal glial cell activation might play an early role in the onset of the inflammatory process responsible for retinal damage at later stages of the disease. This hypothesis is further supported by the reported elevated levels at this stage of the disease of the adhesion molecules ICAM-1 and the neutrophil chemotactic MCP-2, molecules also produced by MGCs and involved in leukostasis, another phenomenon observed early in DR pathology and associated with inflammation [48,49,50].

Various inflammatory cytokines—IL-1β, IL-6, IL-8, TNF-α and MCP-1—have been reported elevated in ocular tissues from non-proliferative DR (NPDR) patients. One study detected even higher levels of IL-8 and TNF-α in diabetic eyes with NPDR than with active PDR [51]. The increase in these cytokines produced by activated microglia, endothelial cells, macroglia, and later even neurons, highlight the increased activity of these inflammatory cytokines in the early stages of DR and the progression of the inflammatory response throughout all cell types of the retina [52]. The accumulation of these inflammatory mediators has been proposed to contribute to early neuronal cell death in the retina in DM. Some of the cytokines identified, such as MIP-1, IL-1 and IL-3, are thought to also have a role in angiogenesis, as established in experimental ischemic mouse models suggesting that inflammation also contributes and precedes the development of neovascularization in PDR [21,53,54]. Significantly higher vitreous levels of neurotrophins (NTs), a group of specific growth factors, such as nerve growth factor (NGF), brain-derived neurotrophic factor (BNDF), NT-3, NT-4, ciliary neurotrophic factor (CNTF) and glial cell line-derived neurotrophic factor (GNDF) could be detected in DR patients, with even higher levels in NPDR compared to PDR [51]. This might be an attempt by glial cells such as MGCs to rescue compromised neurons during this early stage of DR.

In patients with PDR, increased vitreous concentrations of the aforementioned cytokines and neurotrophins along with other growth factors such as VEGF, platelet-derived growth factor (PDGF), insulin-like growth factor (IGF-1), basic fibroblast growth factor (bFGF) and hepatocyte growth factor (HGF) have been reported [55]. Of note, analysis of vitreous samples from PDR patients also revealed increased levels of soluble cytokine receptors, such as sIL-2R [56]. This increase constitutes a known negative regulatory mechanism of cytokine signaling, suggesting that counter-regulatory mechanisms of angiogenesis and inflammation exist within the eye. The development of PDR is a multistage event, including angiogenesis, in which basement membrane degradation, endothelial cell migration and proliferation, followed by capillary tube formation, occur. Matrix metalloproteinases (MMPs), which are important regulators of those migratory and tissue remodeling events, have been reported to be upregulated in PDR as well [57].

In patients with DME, levels of angiopoietin-2 (Ang-2), an important modulator of angiogenesis, are significantly elevated along with inflammatory cytokines and VEGF [12,58]. Currently there are no widely accepted valid biomarkers to monitor DR severity or efficiently classify patients for optimal assessment of treatment efficacy, but reports indicate, that VEGF, HGF, IL-6 and MCP-1 intravitreal concentration increase with the progression of DR from the non-proliferative form to active PDR [55,56]. Furthermore, IL-6 levels have been shown to positively correlate with retinal macular thickness [59].

A variety of the inflammatory mediators mentioned in the previous paragraphs are activated in DR, but the signaling involved in initiating this response is less clear. One of the caveats of using vitreous or aqueous humor levels to assess the expression of proteins associated with diabetes and DR, is the notion that the changes observed might just represent changes in circulating serum levels. Of note, it has been demonstrated, that the total vitreous protein content does not differ between NPDR and PDR patients [51], suggesting that increased vitreous protein levels present a real increase in secretion rather than just leakage of serum proteins into the vitreous because of a disrupted BRB.

Nevertheless, many features of inflammation including leukostasis, neutrophil and macrophage infiltration, complement and microglial activation, upregulation of cytokines, increased blood flow, vascular permeability and tissue edema have been described in animal models and human patients with DR [49,60,61,62,63,64]. Furthermore, inhibition or deletion of pro-inflammatory molecules has been demonstrated to dampen diabetes-induced vascular and neurodegenerative pathology in animal models of DR [65,66,67]. Thus, in the next sections we breakdown the specific contribution of inflammation to the main hallmarks of DR: angiogenesis and neurodegeneration.

### 3.3. Contribution to Vascular Pathology

Evidence of a chronic low-grade inflammation of the retina of patients with advanced DR raised the question of its involvement in the development of edema and pathogenic vascularization [95,96]. Upregulated pro-inflammatory cytokines in the diabetic environment may directly induce vessel formation via engagement of target endothelial cells and indirectly, by inducing endothelial cells to produce pro-angiogenic mediators [97,98,99]. Endothelial cells are extremely susceptible to cytokines, especially IL-1β, TNF-α, and IFN-γ, which then induce production of endothelial cell–derived cytokines IL-8, MCP-1, and Regulated and normal T cell expressed and Secreted (RANTES). In vitro studies demonstrated that endothelial cells respond to cytokines rather than high glucose for induction of inflammatory pathways and apoptotic changes, suggesting that diabetes–related endothelial injury is primarily due to glucose-induced cytokine released by neighboring cells rather than a direct effect of hyperglycemia on endothelial cells themselves [100]. Upon cytokine stimulation they also secrete intracellular adhesion molecules such as ICAM-1 and VCAM-1, resulting in attraction of leukocytes, a process called leukostasis [101]. Indeed, animal studies have shown that 4 weeks after diabetes induction by streptozotocin-injection, before any vascular defects can be recorded, leukocytes start to adhere to the retinal capillaries with subsequent migration into the retina [102,103]. As a result, it has been proposed that the vascular wall integrity diminishes leading to increased vascular permeability. This allows extravasation of vascular fluid and migration of additional immune cells (neutrophils, monocytes) into the tissue. Concurrently, loss of capillary pericytes causes endothelial cell degeneration, which has been associated with accumulation of vacuoles and debris in the basement membrane resulting in its thickening, and ultimately vascular lumen occlusion [104,105,106,107].

The resulting retinal ischemia and hypoxia are strong stimulus for endothelial and glial cells to promote the expression of VEGF and other pro-inflammatory cytokines such as TNF-α, IL-6, and IL-1β, an effect which is in part mediated trough activation of the transcription factor hypoxia-inducible factor 1 (HIF-1) in the cells [108,109]. As inflammation increases in the diabetic retina it does so in a self-propagating manner. Indeed, among the ischemia-induced factors are chemokines such as MCP-1, responsible for attracting macrophages into less perfused areas [110]. Hypoxia-activated macrophages, glial cells and microglia cells produce TNF-α and IL-6 and other inflammatory cytokines and growth factors, which in turn stimulate further release of MCP-1, IL-6 and VEGF by endothelial cells, potentially resulting in increased vascular permeability as seen in NPDR and DME [21,110].

Angiogenic responses of endothelial cells are regulated by inflammatory cytokines and growth factors as well, resulting in the development of new vessel in PDR. The importance of inflammation for this phenomenon has been demonstrated by the fact that blocking inflammation reduces neovascularization. In a murine model of choroidal neovascularization, inhibition of monocyte recruitment by deleting MCP-1 [111] or deletion of ICAM-1 or CD18 led to significant inhibition of neovascularization [112]. Similarly, inhibition of Cyclooxygenase-2 (COX-2), which normally generates prostanoids, reduced the production of VEGF and subsequently vascular leakage in an animal model of DR. It also reduced retinal neovascularization in rodent models of ischemic proliferative retinopathy [113,114]. In addition, High-mobility group box-1 (HMGB1), a DNA-binding protein which facilitates gene transcription and which can be actively secreted by activated monocytes, macrophages and retinal endothelial cells has been linked to inflammation and angiogenesis in DR [115]. Extracellular HMGB1 functions as a pro-inflammatory cytokine and induces the expression of adhesion molecules and other cytokines and chemokines [116,117]. It also directly induces vessel formation by targeting endothelial cells or indirectly by induction of pro-angiogenic factors in endothelial cells and leukocytes [115]. Furthermore, VEGF itself also serves as pro-inflammatory molecule by promoting the expression of other pro-inflammatory cytokines such as I-CAM, MIP-1α, MCP-1 and IL-8 [60,118,119,120]. A role for inflammation in the development of pathological retinal neovascularization in DR was also seen in the rat model of oxygen-induced retinopathy (OIR), which utilizes hypoxia to generate neovascularization and capillary non-perfusion, thus mimicking what it seen in human patients with PDR. Gene expression of NF-κB and protein expression of IL-6 and TNF-α were significantly increased in the retinas of OIR rats compared to controls [121]. Taken together, studies in the OIR models strongly support an interconnection between vascular alterations and inflammation, not only in the early stages of DR, but also in later stages of the disease, including neovascularization and edema.

### 3.4. Contribution to Neurodegeneration

In addition to the vascular perturbations, the neurosensory retina is profoundly altered in diabetes. An emerging issue in DR research is the focus on the precise relationship between inflammatory alterations and the loss of neuronal function.

VEGF, which is a mediator of inflammation and angiogenesis, positively affects neuronal growth, differentiation and survival [122]*.* In vitro VEGF stimulates axonal outgrowth, improves the survival of ganglion neurons, and can rescue cerebral neurons from apoptosis induced by serum withdrawal. In vivo VEGF coordinates migration of motor neuron soma and local delivery of VEGF prolongs motor neuron survival [123]. The receptors for VEGF are present in normal retinal neuronal cells, indicating a possible functional role for VEGF in the neural retina [124]. Gene expression studies in the brain and retina also suggest that VEGF is upregulated by hypoxia preconditioning, a brief ischemic episode that protects neurons, against subsequent prolonged ischemia-reperfusion-related damage [123]. Aside from VEGF, insulin is another obvious pro-survival factor involved in diabetes and an interesting target to promote retinal neuron survival. Indeed, several groups have demonstrated that systemic and local administration of insulin can rescue retinal neurons from cell death in the diabetic rat retina [24,125]. Other neuroprotective factors include neuroprotectin D1 (NPD1) [126], brain-derived neurotrophic factor (BDNF) [127], GDNF [128], CNTF [129] and NGF [130], all of which have been suggested to be involved in the neurodegenerative and inflammatory process that occurs in DR. Early on, increased cytokine/growth factor expression may serve an adaptive function to maintain neuronal function but, over time, becomes maladaptive, at which point the role of VEGF and other factors is impaired and can lead to alteration of the BRB and induction of inflammatory responses.

Evidence further indicates that inflammatory processes themselves are activated in retinal neurons in response to diabetes. Hyperglycemia has been demonstrated to induce a pro-inflammatory phenotype in retinal neurons and their supporting glia in vitro [131,132]. While cytotoxic effects of high glucose treatment itself cannot be completely excluded, it has been demonstrated, that also in vivo hyperglycemia increased circulating cytokine concentrations by an oxidative mechanism in diabetic patients [133]. This suggests a causal role for hyperglycemia in the immune response associated with diabetes. In addition, immunohistochemical studies demonstrated translocation of NF-κB subunits into nuclei of retinal neurons in STZ-induced diabetic rats, suggesting that this pro-inflammatory transcription factor is also activated in neurons during diabetes [134]. Recent reports further provided evidence, that photoreceptors contribute to the inflammatory response in DR [135]. They are highly oxygen demanding cells and hence have shown to be the main sources oxidative stress in the retina in DR [136]. Moreover, it has been demonstrated, that photoreceptors themselves produce inflammatory cytokines such as ICAM-1, COX-2 and iNOS in experimental diabetes. They further release soluble factors in vitro that stimulate TNF-α production in leukocytes and endothelial cells, contributing to the vascular cell death in DR [137].

Consequently, pharmacologic interventions specifically targeting inflammation have been shown to reduce diabetes-induced neurodegeneration in animals studies and patients with DR. Yang et al., demonstrated that treatment with baicalein, a herbal medicine compound, suppressed diabetes-induced inflammatory cytokine upregulation, leading to reduced vascular leakage and ganglion cell loss in the retinas of diabetic rats [138]. The neuroprotective actions of the tetracycline derivative minocycline are mediated by the inhibition of microglial activation and of caspase-3 activation in diabetic rats [21]. Furthermore, doxycycline, another tetracycline derivative was able to prevent neuronal function loss, assessed by frequency doubling perimetry, in patients with NPDR [139].

Despite the above success, other studies have demonstrated that diabetes associated neurodegeneration is not exclusively due to inflammation. Cluster of differentiation (CD) 40, a TNF receptor, which is expressed on various hematopoietic and non-hematopoietic cells, is expressed by MGCs in the retina [140]. Interaction of CD40 with his ligand CD154 not only regulates cellular and humoral immunity [141], but also activates inflammation by activating microglial cells [142]. Of note, inhibition of microglial activation in diabetic CD40 knockout (KO) mice prevented capillary degeneration, but despite a suppressed inflammatory response, the mice maintained ERG b-wave defects [143]. Similarly, diabetic mice deficient in nitric oxide generating inducible nitric oxide synthase (iNOS) did not develop the expected degeneration of retinal capillaries, leukostasis as well as ganglion cells loss, however they still presented with retinal functional impairment recorded by ERG [144]. More recently, inhibition of the receptor for advanced glycation end products [36] signaling pathway, which has been demonstrated to induce inflammatory changes in DR [145], did not protect from vision loss in diabetic animals [146]. These studies strongly support the notion, that inflammatory processes contribute, but not cause the development of DR. In this context other pathways such as oxidative stress have been shown to be important in the development of DR [147].

Furthermore, the initiation and regulation of inflammation is a considerably complex mechanism, e.g., many cytokines are known to activate NF-κB and other pro-inflammatory mediators; thus, inhibiting one component of this cascade will only be partially effective.

Nevertheless, from a therapeutic point, it is likely that identifying and halting key steps of the inflammatory process contributing to tissue destruction will help to control retinopathy. A more in-depth analysis of the role of inflammation in the neuro-glial crosstalk in DR will be discussed thereafter.

## 4. Role of Glial Cells in Diabetic Retinopathy

**Figure 3:**
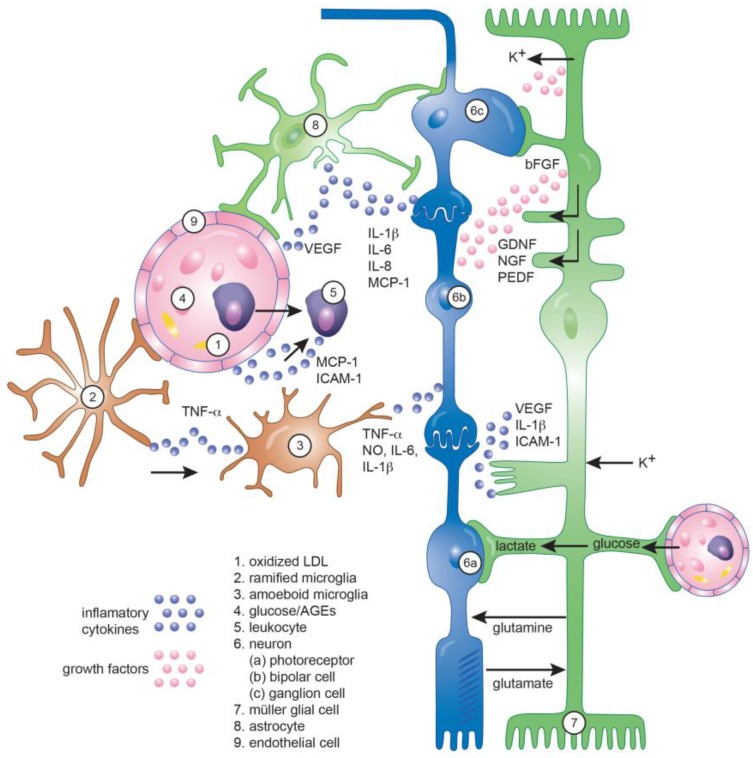
Schematic of a mammalian retina depicting the interactions between glia, neurons and endothelial cells with special regard to the inflammatory chemokines involved. Blood vessels and endothelial cells in pink (**9**), leukocytes in purple (**5**), macroglial cells in green (**7**,**8**), microglia in brown (**2**,**3**) and neurons in blue (**6a**–**c**). Scheme showing kalium homeostasis, glutamate metabolism and the secretion of trophic factors, chemokines and interleukins. AGEs, advanced glycation end products.

The two main retinal glial cells are Müller glial cells (MGCs) and astrocytes. Each cell type differs markedly in distribution, morphology, and function. Together with microglia, the main resident immune cells in the retina, they not only provide structural support, but they are also involved in maintaining the complex homeostasis of the retina by regulating the metabolism, the phagocytosis of debris and the cycling of neurotransmitters and trophic factors (Figure 3).

One of the first signs of inflammation in DR is the activation of glial and microglial cells. The conventional view is that microglia are the first responders, initiating an inflammatory response that participates to triggering MGCs gliosis [148]. Early histopathological studies reported that both macroglial and microglia cells show early structural changes after 4 weeks of experimental diabetes in rats. These studies originally suggested that diabetes affected the density of microglia and MGCs and that all glial cells sense metabolic changes similarly and at the same time [61]. However, most recently, several studies in animal models and post-mortem human tissues have suggested that the rapid microglial activation and its typical morphological changes can be detected prior to macroglial activation [149]. This notion is also supported by additional histopathologic studies showing that many inflammatory molecules, such as TNF-α, can be detected early in the diabetic retina, often in association with microglia [138]. However, using mice with CD40 expression restricted to MGCs, Portillo et al., showed that CD40 in MGCs is sufficient to upregulate retinal inflammatory markers by microglia in experimental diabetes [142], supporting that the activation of microglial cells is, at least in part, dependent on other cell types of the retina. Alternatively to the CD40 dependent activation pathway, factors such as free fatty acids that are released by compromised neurons represent another mean for initial microglial activation [149]. Of note, other retinal cell types such as endothelial cells, astrocytes and neurons can respond to the initial activation of microglial cells by themselves participating and amplifying the inflammatory response. A support for this theory comes from the observation, that in vitro astrocytes require microglial presence to respond to inflammatory stimuli [150].

Early glial cell activation benefits the stressed retina, since activated glia phagocytose apoptotic cells, clear debris and cytotoxins, and secrete neurotrophic factors [151]. However, chronic gliosis in DR is detrimental, as activated glia may not properly regulate retinal blood flow or maintain the BRB. Moreover, gliotic cells secrete inflammatory cytokines, cytotoxic molecules and VEGF perpetuating both, vascular dysfunction and neurodegeneration [151].

### 4.1. Microglia

Microglia cells are the resident immunocompetent cells in the central nervous system. The most reliable characteristics differentiating microglia from invading monocytes are a low CD45 expression and a highly ramified, dendritic morphology [152]. In the developing retina, microglia are involved in the pruning of neuronal and vascular networks through the phagocytic removal of apoptotic cell debris [31,153]. In the adult retina, ramified (‘‘resting”) microglia reside in the inner and outer plexiform layers and they are an important source of neurotrophic factors that support neuronal survival. Resting microglia continuously monitor their environment and when activated, they shift towards an amoeboid morphology [154].

Although the precise mechanisms of glial activation in DR are not yet fully understood, it is likely that both genotypic variations and genetic susceptibility regulate individual microglial response in the course of DR [148]. Microglia, with their highly motile processes extending into the capillary wall, are assumed to be, together with Müller glia, the first detector of metabolic changes in diabetes [155]. They sense dyslipidemia, increases in ROS and cytokines, and the accumulation of AGEs. Amadori-glycated albumin, an AGE, was shown to specifically stimulate microglia to produce TNF-α in early stages of DR [156]. Once activated, microglia become mobile and migrate to the site of inflammation and will produce a wide range of pro-inflammatory cytokines, glutamate, ROS, nitrous oxide (NO) and proteases. Under chronic activation conditions, this cocktail can be extremely toxic to retinal ganglion cells, greatly exacerbating neuronal cell dysfunction [138,149]. Indeed, prolonged activation of microglia is associated with high levels of glutamate, iNOS and pro-inflammatory cytokines such as IL-1β, IL-6, IL-12 and TNF-α which can culminate in the activation of caspases, exacerbating retinal neuron cell death retina [157].

Case studies exclusively using histological techniques on retinal cross-sections revealed increasing numbers of moderately hypertrophic microglial cells in the plexiform layers of NPDR patients [158]. They further showed that in tissues from PDR patients, clusters of microglial cells could be found surrounding ischemic areas and new vessels, while suggesting an even more significant rise in their total number [149].

### 4.2. Müller Glial Cells

Müller glial cells are the major glial cell type in the mammalian retina, representing 90% of the retinal glia. They form the supporting architectural structure radially stretching across the entire thickness of the neuroretina and participate in forming the outer and inner limiting membranes [159]. Müller glial cells also strongly link with retinal vessels and with the RPE, which establish connections to the subretinal space and choroidal vasculature [160]. Since Müller cells have contact to virtually every cell type in the retina they are uniquely positioned to perform a wide variety of functions necessary to maintaining retinal homeostasis. In the healthy retina, they recycle neurotransmitters, including glutamate thus preventing excitotoxicity; redistribute ions by spatial buffering, participate in the retinoid cycle, and regulate nutrient supplies [160,161]. Any disturbance to the retinal environment will influence Müller cells, which in turn will affect the entire retina [29].

Müller glia cells are deeply involved in DR, showing morphological and functional alterations from the early phases of the disease. The most striking example being that their size significantly increases in rodent models of diabetes, while a progressive increased expression of the stress marker GFAP becomes prominent in tissues from both animal models and diabetic donors [61].

In addition to GFAP upregulation, Müller cells acquire a complex and specific reactive phenotype, which is also characterized by induction of acute-phase response proteins and other inflammatory related genes [22]. In vitro studies have provided compelling evidence that Müller cells are a significant source for many growth factors and cytokines, when stimulated with stress conditions, including elevated glucose levels [132]. Considering that most of the growth factors, cytokines, and chemokines released by Müller cells have been identified in the vitreous of diabetic patients, it has led to the hypothesis that Müller cells contribute to the overall synthesis of these factors in vivo [162]. Non-targeted discovery approaches assessing the gene expression profile of Müller cells after 6 months of STZ induced diabetes, revealed that among 78 altered genes, one third are associated with inflammation, including complement factors, VEGF, ICAM-1 and IL-1β [163]. Of note, some of these cytokines in turn have been shown in other studies to stimulate production of other cytokines by Müller glia, suggesting a possible amplification effect. [164].

Taking into account their central role in maintaining the homeostasis in the retina and their contribution to the inflammatory response in DR, understanding Müller cell functions within the retina and restoring such function in DR could become a cornerstone for developing effective therapies to treat diabetic retinopathy.

### 4.3. Astrocytes

Like Müller cells, astrocytes are connected to retinal blood vessels and neurons and play a critical role in maintenance of the BRB, including regulation of blood flow [165]. Astrocytes also provide energy substrates to neurons and regulate the production of trophic factors and antioxidants. In contrast to Müller cells, they are largely restricted to the nerve fiber layer and ganglion cell layer. The distribution of retinal astrocytes is strikingly correlated with the presence and distribution of retina blood vessels, so vascularized areas of the retina are rich in astrocytes, while avascular zones such as the fovea, contain no astrocytes [166]. In response to injury or disease, they have been shown to upregulate the expression of various genes encoding cytokines, chemokines and elements of the complement cascade. Such response has been suggested to participate in compromising the integrity of the BRB and promoting retinal degeneration [167].

In response to the dysmetabolic and hypoxic diabetic environment, astrocytes become activated, also called reactive, and produce a variety of pro-inflammatory cytokines, such as IL-6, IL1β, IL-8, COX-2, transforming growth factor-α (TGF-α), epidermal growth factor (EGF), macrophage inflammatory protein 2α (MIP-2α) and VEGF [168,169]. Apart from inflammatory cytokines reactive astrocytes can secrete chemokines, including MCP-1, C-C motif chemokine ligand (CCL) 5, CCL20, C-X-C motif chemokine ligand (CXCL) 10, CXCL12, CXCL1, CXCL2, and CX3CL1 [170]. These chemokines are involved in the recruitment of microglia, monocytes/macrophages, T-cells, and dendritic cells, amplifying the inflammatory response. Over time astrocyte number seem to decrease in DR patients, as demonstrated by decreased GFAP expression, which then disrupts the inner BRB, contributing to vascular leakage in the retina [61].

### 4.4. Role of Inflammation in the Neuro-Glial Crosstalk in DR

Glial cells and neurons are intimately associated to make-up the neuro-glial unit. Glial cells are the interface between the neurons and the vasculature and are thus key regulators of neuronal function. Müller glial cells convey circulating glucose into the retina for ATP production and provide intermediary compounds such as lactate to neurons [64]. They play multiple key roles for retinal tissue homeostasis, from storing glycogen for conversion to lactate, to synthesizing retinoic acid from retinol, regulating extracellular ion concentrations to modulate plasma membrane polarization/depolarization, participating with neurons in the glutamate/glutamine cycle to control neurotransmission, and protect neurons from glutamate excitotoxicity [171]. Another important function of macroglial cells is to redistribute the neuronal metabolic by-products such as carbon dioxide and water from glucose metabolism, but also potassium and neurotransmitters associated with neuronal activity, into the blood and vitreous [161]. MGCs maintain proper retinal function by participating in a process known as ‘‘potassium spatial buffering”, a process that redistributes and normalizes K^+^ in the surrounding microenvironment to avoid prolonged accumulation of K^+^ [172]. This response is coupled to the regulation of water homeostasis by the glial water channels aquaporins (AQP) such as AQP4 [173]. Macroglial cells, besides supporting the synaptic activity, maintain the integrity of the BRB and regulate the vasoconstriction related to neuronal activity, a process named neurovascular coupling by the release of neuroactive and vasoactive substances [174]. Macroglial and microglial cells also regulate the survival of neurons by a molecular network made up of several neurotrophic factors (BNDF, CNTF, GDNF; NGF, NT-3, and bFGF) [148]. Microglial and macroglial cells are further responsible for cellular debris phagocytosis and response to immunological damage [175]. They also play an active role engulfing synaptic material in a process termed synaptic pruning and referred to as the innate surveillance function of resident microglia [176,177].

Diabetes also affects the neuro-glial unit by disrupting the communication between neurons and glia. In DR, accumulating evidence over the past decades have revealed that dysfunctional neuro-glial crosstalk, in part associated with inflammation, plays a critical role in the early course of the disease [138,178]. One of the first altered structural alterations in experimental and human DR is reactive gliosis of macroglial cells, mostly in the initial stages of DM [61]. Reactive gliosis may be interpreted as an effort to limit the extension of tissue damage but sustained inflammation in DR lead to the more severe forms of reactive gliosis [179]. Macroglial cells undergo further hypertrophy, lose their functionality, ultimately forming glial scars that are inhibitory to axonal regeneration and neuronal survival [162,180].

Microglial activation is the main mechanism by which neuroinflammation starts in response to nervous tissue perturbations [15]. Microglial activation in DR acts as a double-edged sword, as its primary function is neuroprotection. On the other hand, activated microglia produce pro-inflammatory mediators such as IL-1β, IL-3, IL-6, TNF-α, VEGF, lymphotoxin, MIP-1α, MMPs, NO, ROS and complement factors, primarily promoting neuronal cell death when sustained. [138,149,157]. The mechanism by which cytokines contribute to neural apoptosis is not clear but may involve the induction of excitotoxicity, oxidative stress or mitochondrial dysfunction [181,182,183]. A representative example of a neuron-microglia interaction is the FKN/CX3CR1 system, which provides a mechanism to modulate microglial activation by neurons. Fractalkine (CX3CL1 or FKN) exists on neuronal membranes and functions by signaling through its unique receptor CX3CR1 present on microglia. Several reports support the notion that FKN exerts an inhibitory signal on microglia [184]. It has been demonstrated, that increased inflammatory microglial responses in absence of the fractalkine receptor contribute to inflammatory-mediated damage to neurons in the diabetic retina [65]. Accordingly, microglial receptor proteins appear as important targets for the development of drugs to prevent or manage retinal neuroinflammation.

Next to microglia, astrocytes react becoming activated during diabetes and undergo a series of changes as mentioned earlier such as secretion various cytokines, chemokines, MMPs and elements of the complement cascade, contributing to neuronal degeneration [138,167,168].

Notably, Müller cells exposed to hyperglycemic conditions also produce increased levels of pro-inflammatory molecules such as IL-1β and TNF-α, which then induce expression of pro-death cytokine IL-8 [164]. Similar mechanisms have been proposed for other cytokines secreted by Müller cells such as IL-6, NO and COX-2 [132].

Water accumulation in retinal neurons and glial cells is another pathogenic factor involved in retinal degeneration during DR. Müller cells adapt their morphology to the increased size of activated retinal neurons by extending and thinning the inner stem process. The water flux through AQP4 is involved in the rapid volume regulation of retinal Müller cells in response to osmotic stress. Diabetes is known to enhance the amount of retinal expression of AQP4 and down-regulation of AQP4 exacerbated experimental DR through increased expression of pro-inflammatory factors [185]. Therefore, regulation of retinal function by AQP4 may attenuate diabetic retinopathy, offering a promising therapeutic strategy for diabetic retinopathy [185].

Neurodegeneration leads to the alteration of expression of growth factors (e.g., VEGF) by glial cells [186]. The release of VEGF by Müller cells under conditions stimulating gliosis is especially interesting, as it acts in two directions. VEGF signaling in Müller cells may represent a stress-responsive neuroprotective mechanism, particularly under hypoxic conditions. VEGF receptor 2 (VEGFR2)-mediated signaling in MGCs has been also shown to stimulate the production of BDNF and GDNF [178]. As a result, the loss of VEGFR2-mediated signaling in MGCs caused a significant elevation of apoptotic MGCs and neurons during experimental DR [178]. On the other hand sustained VEGF release also acts as a pro-inflammatory molecule by inducing the expression of other cytokines, thus exacerbating retinal neurodegeneration [187].

Conversely a growing body of evidence indicates that hyperglycemia-induced downregulation of a number of important neurotrophic factors, such as NGF, Pigment epithelium-derived factor (PEDF), CNTF, interphotoreceptor retinoid-binding protein (IRBP) and somatostatin, the loss of which may have a direct impact on neuronal survival [188]. Factors, such as PEDF, functions as an anti-inflammatory and anti-oxidative agent by inhibiting the activation of the NF-kB and Extracellular signal-regulated kinase 1 (ERK1) pathways. When secreted by Müller cells, this factor acts directly on PEDF receptors at the surface of RGCs [189,190]. PEDF levels were found to be decreased in cultured MGCs subjected to hyperglycemia and mild hypoxia [191,192]. However, under severe hypoxic conditions, PEDF was upregulated, potentially reflecting an attempt to rescue retinal neurons [192]. Why this observation does not translate in vivo, where decreased vitreous levels of PEDF are demonstrated also at later stages of DR such as PDR is not clear yet [83]. CNTF has been shown to directly prevent photoreceptor apoptosis and indirectly rescue neurons by stimulation of Müller cells to produce photoreceptor survival factors.

Altogether, studies clearly show how modulation of the inflammatory response by glial cells might act as an important target to prevent early neuronal damage in DR.

## 5. Emerging Trends in Inflammation-Related Mediators of Diabetic Retinopathy

### 5.1. Alpha-Crystallins

The two small heat shock proteins α-crystallins, αA and αB, [193,194] have been widely studied for their roles in the lens, but we are just starting to understand their function in the retina. Recent studies revealed that α-crystallins are rapidly upregulated in models of acute retinal injury, including light toxicity and trauma, suggesting that α-crystallins play a role in the retina’s response to cellular stress and damage [195,196,197]. We recently demonstrated that these chaperones were highly expressed in both RGCs and Müller glial cells of diabetic animals and human patients with DR [198]. Of note, αA-crystallins not only inhibit pro-apoptotic pathways in retinal neurons, but also act as a secreted pro-survival factor [198] and modulator of the expression of inflammatory molecules by glial cells (unpublished data), for the first time establishing a direct link between α-crystallins and neuro-inflammation. Consistent with a critical role in glial cells, α-crystallins have been reported to be upregulated in astrocytes of patients with Alexander disease and microglial cells of patients with Alzheimer’s disease [199,200,201]. It has been hypothesized that the increased expression of αB-crystallin is part of the normal stress response to regulate aberrant protein aggregation and may prevent apoptosis in the face of the increased phagocytosis and protein aggregate load [202]. Overall, α-crystallin chaperones affect microglial and astrocytic activation in an anti-inflammatory manner resulting in decreased cytokine production and tempering of the innate immune response. Further research, on the protective function of αA-crystallin should govern the development of specifically engineered crystallin proteins, which could be applied to the treatment of diabetic retinopathy and other neurodegenerative conditions.

### 5.2. Matrix Metalloproteinases

Matrix metalloproteinases constitute a large family of secreted and membrane associated zinc-dependent proteinases that degrade at least one component of the extracellular matrix (ECM). They are known to execute functions in motility, cell growth, injury response, and remodeling of the ECM by both, degrading it and controlling proteolysis of specific targets including cytokines, growth factors and adhesion molecules [203,204]. MMPs are highly sensitive to oxidative stress in the diabetic environment, with MMP-2 and MMP-9 being induced by increased ROS [57,205,206]. It has been suggested that these MMPs, especially MMP-9, can increase the inflammatory response by degrading some of the major components of the basement membrane, enabling invasion of the injured tissue by circulating immune cells. MMP-9 knockout-mice indeed showed significant reductions in the expression of inflammatory markers such as MIP-1α, MIP-1β, and MCPs after spinal cord injury [207]. In addition, activated MMPs also contribute directly to angiogenesis and the increase in vascular permeability in DR by proteolytic degradation of occludin, disruption of the tight junction complex [57,208], and increased tissue levels of VEGF [209], making them interesting potential therapeutic targets.

### 5.3. Toll-Like Receptors

Toll-like receptors are pathogen-associated molecular pattern receptors that have been found on the surface of all cell types in the retina. Their activation results in the production of cytokines/chemokines in an NF-κB dependent manner [210]. Among the various TLRs, TLR-2 and TLR-4 have been most consistently associated with type 1 and type 2 diabetes. For example, genetic polymorphism in the TLR-4 gene is strongly associated with insulin resistance levels in patients with type 2 diabetes [211]. Additionally, TLRs also promoted the secretion of angiogenic growth factors in arterial endothelial cells [212] and led to choroidal neovascularization in vivo [213]. Human microvascular retinal endothelial cells demonstrated increased TLR-2 and TLR-4 expression when exposed to high glucose, an observation also associated with increased expression of pro-inflammatory molecules. Supporting a regulatory role of TLRs in this process, this effect was blunted by using a TLR4/2 inhibitory peptide or antioxidant treatment [214].

### 5.4. Complement System

While diabetes is not considered a complement-mediated disease, some data suggest a link between DR progression and complement dysregulation. Evidence for such a link comes from the observation of increased expression of several complement factors in the vitreous of patients with DR. Among over 30 small proteins and protein fragments identified as part of the complement pathway, some of the key regulators have been reported increased in PDR patients: C3, C4b, C9, and factor B (CFB) [215,216]. C5 cleavage, along with C3, is considered one of the two critical steps for the full activation of the complement pathways. A fully activated complement system results in the formation of the membrane attack complex (MAC or C5b-9) that can kill pathogens but also under certain conditions host cells, thus could play a role in neurodegeneration and vascular permeability. Additionally, cleavage products C3a and C5a have a chemotactic effect on neutrophils, mast cells, and lymphocytes, potentially exacerbating the inflammatory response. Finally, Müller glial cells constitutively express C5aR, the expression of which is further increased by high glucose exposure, and associated with release of IL-6 and VEGF by these cells, a mechanisms that could be involved in promoting the overt-inflammatory response observed in diabetes [217].

## 6. Therapeutic Concepts in Diabetic Retinopathy

### 6.1. Current Therapies

Several therapeutic approaches are in use clinically to inhibit the development or progression of DR. Metabolic control (tight glycemic control, regulation of blood pressure and hyperlipidemia) is associated with a lower risk of retinopathy [218,219]. Specific therapeutic options for DR include laser coagulation, surgery and intravitreal injection of anti-VEGF agents. Alternatively, intravitreal injections of steroids have been shown to reduce vascular permeability, reduce the breakdown of the BRB, inhibit leukostasis, inhibit VEGF gene transcription and translation [220]. Because of an increased rate of elevated IOP and cataract formation with steroids, the use of intravitreal steroids in clinical practice is currently reserved as the second-line treatment in patients with previous cataract surgery [221].

Unfortunately, all these therapeutic interventions have significant side effects and are only useful for patients with more advanced stages of DR, when vision is already compromised, and neurodegeneration already occurred. Considering the role of inflammation in the pathogenesis of DR, inhibiting the inflammatory pathway has been an appealing treatment option for DR in future practices.

### 6.2. Inflammation-Targeting Therapies under Development and Validation

#### 6.2.1. Nonsteroidal Anti-Inflammatory Drugs (NSAID)

Clinically the beneficial effect of NSAIDs, that inhibit COX enzyme-mediated ICAM-1 expression and leukostasis [222,223], has been suggested in a study reporting reduced incidence of DR in patients taking salicylates for the treatment of rheumatoid arthritis [20]. This finding encouraged the launch of clinical trials, which showed the development of retinal microaneurysms is only significantly attenuated in patients with early stage of DR when treated with high dose of aspirin (900 mg/day) [224,225]. Alternatively, while systemic administration of specific COX-2 inhibitors is made impossible by increased incidence of heart attacks and strokes [226], preclinical studies with topical administration were shown to reduce signs of DR [227,228].

#### 6.2.2. Blocking Inflammatory Molecules

The effect of anti-TNFα therapy has been studied in a few clinical cases. Patients with refractory DME treated with intravitreal Etanercept (TNFα inhibitor) showed no statistically significant improvement [229]. Infliximab, a TNFα neutralizing antibody on the other hand improved visual acuity and reduced macular thickness in 4 patients who failed to improve in response to laser photocoagulation treatment [230]. Larger trials are needed to determine the efficacy of these drugs in DME patients.

Alternatively, a pilot study on the efficacy and safety of a selective IL-1β antibody, Canakinumab (Novartis, Basel, Switzerland), for the treatment of patients with proliferative DR showed stabilization but no regression of retinal neovascularizations, along with promising effects on diabetic macular edema reduction [231]. Similarly, a clinical trial in DME patients comparing an oral inhibitor targeting the receptors for MCP-1, CCR2/CCR5 (Pfizer, New York, NY, USA) to intravitreal anti-VEGF therapy showed non-inferiority of the study drug regarding gain in visual acuity and reduction in central retinal thickness [232].

Leukostasis, one of the early steps of inflammation, is dependent on specific integrins on the endothelium to trigger leukocyte adhesion and infiltration [233]. The DEL MAR Phase 2b trial using an integrin antagonist, Luminate, ALG-1001 (Allegro Ophthalmics, San Juan Capistrano, CA, USA) as sequential therapy to a single anti-VEGF injections in DME patients compared to anti-VEGF monotherapy reported sustained and equal visual acuity gains for both treatments [234].

#### 6.2.3. Inflammatory Growth Factors

Angiopoietins represent a family of inflammatory growth factors that bind to the receptor tyrosine kinase Tie2 and are important modulators of angiogenesis. A recent study also reported that Ang-2 promotes adhesion of monocytes and the subsequent tissue inflammation by modulating the TNF-α-induced expression of ICAM-1 [235]. Thus, the Ang-2 pathway is currently being targeted in a recent ongoing clinical trial with a Tie-2 activator (AKB-9778, Aerpio Therapeutics, Cincinnati, OH, USA) in patients with NPDR, where improvements in DR severity will be evaluated. A previous study already demonstrated a significant benefit of AKB-9778 combined with anti-VEGF therapy over anti-VEGF monotherapy in the reduction of DME with a trend toward improved visual acuity [236].

#### 6.2.4. The Renin-Angiotensin System (RAS)

Specific blockade of the RAS with the Angiotensin II receptor type 1 (AT1R) blocker (Losartan, Candesartan) and angiotensin-converting enzyme inhibitor (Enalapril) has been shown to reduce the risk of onset and the progression of retinopathy in patients with type 1 and 2 diabetes [44,237,238]. In another clinical trial involving 285 type 1 diabetic patients with normotensive and normoalbuminuria, blockade of RAS with Losartan or Enalapril significantly reduced the progression of retinopathy by 70% and 65% respectively [238]. However, the Diabetic Retinopathy Candesartan Trials which enrolled 3326 patients with type 1 diabetes and 1905 patients with type 2 diabetes, did not observe such a dramatic effect. It has been demonstrated, that Candesartan treatment reduced the incidence of DR but did not prevent progression of DR [237]. Further studies are needed to resolve the discrepancy between the above trials and to fully evaluate the beneficial effect of RAS blockade before its clinical usefulness is fully demonstrated.

#### 6.2.5. Tetracyclines

In addition to their antimicrobial activity, minocycline and doxycycline are known to possess several immunomodulatory and neuroprotective properties [239,240], such as inhibiting the production of NO, cyclooxygenases, prostaglandins, IL-1β and TNF-α and caspases [21,241]. A single-center phase I/II clinical trial in 5 patients with DME showed that minocycline as primary treatment was associated with improved visual function, reduced central macular edema, and vascular leakage [242]. Another clinical trial demonstrated an improvement of perimetric parameters in patients with severe non-proliferative or non-high-risk proliferative DR treated with doxycycline compared to patients who received placebo. Of note, visual acuity and anatomical parameters (such as DR severity levels) were equal among both groups, which may relate to a differential effect of tetracyclines on different stages of DR dysfunction [139].

#### 6.2.6. Photobiomodulation

Photobiomodulation (PBM), also known as low level laser therapy or far-red to near-infrared (FR/NIR) light therapy, consists of series of brief illumination with specific wavelengths of light (600–1000 nm) from a laser or a light emitting diode [243] and results in the activation of signalling pathways within the cell. It has been demonstrated, that it effectively inhibits diabetes induced increase in superoxide production, leukostasis and ICAM-1 expression in STZ induced rats, which resulted in a significant reduction in ganglion cell death and improvement of the photopic b wave ERG amplitude [244]. A subsequent case series by the same group including 4 patients with type 2 diabetes with non-center-involving DME underwent treatment with PBM twice a day for 2–9 months, demonstrated statistically significant decrease in macular thickness by an average of 20%, while the non-treated eye of the same patients featured a slight increase in thickness [245]. Further investigations, using larger patient populations, will be necessary to confirm the efficacy, establish the optimal treatment dose and duration, as well as identify and address potential safety concerns.

## 7. Conclusions

This review summarizes recent clinical and laboratory findings suggesting a pivotal role of inflammation in the pathophysiology of early DR. Many studies carried out in diabetic patients and diabetic animal models have shown that the diabetic milieu provoke increased local expression of inflammatory molecules, such as cytokines, chemokines, and growth factors involved in the development of DR. Glial cells are critically located between vasculature and neurons of the retina, having a key role in closely regulating the retinal microenvironment, which is often responsible for early harm of neuroretina in the beginning stages of DR. Recent findings implicate that these cells also initiate the inflammatory cascade. New technologies able to detect early alterations of the neuroglial unit support the idea that changes in the metabolism of glia cells and subsequent damages of retinal neurons precede microvascular impairment. Additional work has now been performed that suggests that in this manner anti-inflammatory drugs are promising future therapies to address both, vascular alterations and neurodegeneration. However, studies are still needed to better understand the exact molecular mechanisms underlying ocular inflammation in patients with DR.

## Figures and Tables

**Table 1:** Overview over levels of inflammation-related cytokines, chemokines and growth factors in the vitreous or aqueous humor of human diabetic patients with different stages of diabetic retinopathy (DR). If only information on serum levels were available, it is mentioned specifically. ↑ upregulation, ↓ downregulation, ↔ no change

Factors	Diabetic Retinopathy Stage				References
	Diabetics without DR	NPDR	PDR	DME	
AQP4	↑	↑	not known	not known	[47]
GFAP	↑	↑	not known	not known	[47]
VEGF	↑ serum ↔ vitreous	↑	↑	↑	[68,69,70]
IFN-γ	↑	↑	↑	↑	[49,52,71,72]
IL-1α	↑	↑	↑	↑	[49,69]
IL-3	↑ vitreous ↓ serum	↓ vitreous & serum	↓ serum	↑ aqueous no change vitreous	[49,73,74,75]
s-IL2R	↑	↑	↑	not known	[56,73]
MCP-2	↑	↑	not known	not known	[49]
ICAM-1	↑ serum	↑ serum	↑	↑	[68,70,71]
IL-1β	no	↑	↑	↑	[52,76]
IL-6	no	↑	↑	↑	[52,69,70,77]
IL-8	no	↑↑	↑	↑	[51,69,77,78]
IL-2	no	↑	↑	not known	[52,79]
IL-4	no	↑	↑	not known	[52,80]
IL-5	no	↑	↑	not known	[52]
MCP-1	no	↑	↑	↑	[49,77]
TNF-α	no	↑↑	↑	↑	[51,52,70]
sTNF-R	no	↑	↑	not known	[49]
RANTES	no	↑	↑	↑	[49,81]
IP-10	no	↑	↑	↑	[49,77,82]
GM-CSF	no	↑	↑	not known	[49,69]
PEDF	↔ vitreous	↔ serum	↑active PDR ↓ inactive PDR	↓ vitreous	[12,83,84,85,86]
IGF-1	no	conflicting results	↑	not known	[87,88,89]
PlGF	no	no	↑	↑	[90]
IL-10	no	no	↑	↑	[52,69,76]
Complement factors	no	no	↑	↑	[17,63]
bFGF	not known	not known	conflicting results	conflicting results	[89,91]
CD40	no	↑	↑	not known	[92]
HIF-1α	not known	↑	↑	not known	[93,94]

## Abbreviations

AGE	Advanced Glycation End-product
AH	Aqueous Humor
Ang	Angiopoietin
AT1R	Angiotensin II Receptor Type 1
AQP	Aquaporin
BDNF	Brain-derived Neurotrophic factor
bFGF	Basic Fibroblast Growth Factor
BRB	Blood-retinal Barrier
CCL	C-C motif ligand
CCR	C-C receptor
CD	Cluster of differentiation
CNS	Central Nervous System
CNTF	Ciliary Neurotrophic Factor
COX	Cyclooxygenase
CRP	C-reactive Protein
CXCL	C-X-C motif ligand
DR	Diabetic Retinopathy
DCCT	Diabetes Control and Complications Trial
DME	Diabetic Macular Edema
EGF	Epidermal Growth factor
ERG	Electroretinogram
ERK	Extracellular Signal-Regulated Kinase
FDA	US Food and Drug Administration
GDNF	Glial-derived Neurotrophic factor
GFAP	Glial Fibrillary Acidic Protein
GM-CSF	Granulocyte Macrophage Colony-Stimulating Factor
HGF	Hepatocyte Growth Factor
HIF	Hypoxia-inducible Factor
HMGB	High Mobility Group Box
ICAM	Intracellular Adhesion Molecule
IGF	Insulin-like Growth Factor
IFN	Interferon
IL	Interleukin
iNOS	Inducible Nitrous Oxide Synthetase
IP	Interferon γ-induced Protein
IRBP	Interphotoreceptor Retinoid Binding Protein
IRMA	Intraretinal Microvascular Abnormality
IVTA	Intravitreal Triamcinolone Acetonide
LIF	Leukemia inhibitory factor
LPS	Lipopolysaccharide
MCP	Monocyte Chemoattractant Protein
MIP	Macrophage Inflammatory Protein
MMP	Matrix Metalloproteinase
NFG	Nerve Growth Factor
NF-κB	Nuclear factor kappa-light-chain-enhancer of activated B cells
NO	Nitric Oxide
NPDR	Non-proliferative Diabetic Retinopathy
NSAID	Nonsteroidal Anti-Inflammatory Drug
PAMP	Pathogen-associated Molecular Pattern
PDGF	Platelet-derived Growth Factor
PDR	Proliferative Diabetic Retinopathy
PEDF	Pigment Epithelium-derived Factor
PGE	Prostaglandin
PGF	Placental growth factor
PKC	Protein Kinase C
PPAR	Peroxisome Proliferator-Activated Receptor
PPV	Pars Plana Vitrectomy
RAS	Renin-Angiotensin System
RAGE	Receptor for Advanced Glycation End-product
RANTES	Regulated and normal T cell expressed and secreted
RGC	Retinal Ganglion Cell
ROS	Reactive Oxygen Species
RPE	Retinal Pigment Epithelium
SDF	Stromal-derived Factor
sTNF-R	Soluble Tumor Necrosis Factor-Receptor
STZ	Streptozotocin
TGF	Transforming Growth Factor
TLR	Toll-like Receptor
TNF	Tumor Necrosis Factor
UKPDS	United Kingdom Prospective Diabetes Study
VEGF	Vascular Endothelial Growth Factor
